# Polyphenols Mediate Neuroprotection in Cerebral Ischemic Stroke—An Update

**DOI:** 10.3390/nu15051107

**Published:** 2023-02-23

**Authors:** Salaheldin Abdelraouf Abdelsalam, Kaviyarasi Renu, Hamad Abu Zahra, Basem M. Abdallah, Enas M. Ali, Vishnu Priya Veeraraghavan, Kalaiselvi Sivalingam, Larance Ronsard, Rebai Ben Ammar, Devanathadesikan Seshadri Vidya, Palaniyandi Karuppaiya, S. Y. Al-Ramadan, Peramaiyan Rajendran

**Affiliations:** 1 Department of Biological Sciences, College of Science, King Faisal University, Chennai 31982, Saudi Arabia; 2 Department of Zoology, Faculty of Science, Assiut University, Assiut 71515, Egypt; 3 Centre of Molecular Medicine and Diagnostics (COMManD), Department of Biochemistry, Saveetha Institute of Medical and Technical Sciences, Saveetha Dental College & Hospitals, Saveetha University, Chennai 600077, India; 4 Department of Botany and Microbiology, Faculty of Science, Cairo University, Cairo 12613, Egypt; 5 Department of Developmental, Molecular and Chemical Biology, Tufts University School of Medicine, Boston, MA 02111, USA; 6 The Ragon Institute of MGH, MIT and Harvard, Cambridge, MA 02139, USA; 7 Laboratory of Aromatic and Medicinal Plants, Center of Biotechnology of Borj-Cedria, Technopole of Borj-Cedria, P.O. Box 901, Hammam-Lif 2050, Tunisia; 8 Department of Pharmacology & Toxicology, College of Pharmacy, Prince Sattam Bin Abdul Aziz University, Al-Kharj 11942, Saudi Arabia; 9 State Key Laboratory for Conservation and Utilization of Subtropical Agro-Bioresources, Guangxi University, Nanning 530004, China; 10 Department of Anatomy, College of Veterinary Medicine, King Faisal University, Al-Ahsa 31982, Saudi Arabia

**Keywords:** ischemia stroke, oxidative stress, gallic acid, resveratrol, quercetin, kaempferol, mangiferin, epigallocatechin, pinocembrin

## Abstract

Stroke is one of the main causes of mortality and disability, and it is due to be included in monetary implications on wellbeing frameworks around the world. Ischemic stroke is caused by interference in cerebral blood flow, leading to a deficit in the supply of oxygen to the affected region. It accounts for nearly 80–85% of all cases of stroke. Oxidative stress has a significant impact on the pathophysiologic cascade in brain damage leading to stroke. In the acute phase, oxidative stress mediates severe toxicity, and it initiates and contributes to late-stage apoptosis and inflammation. Oxidative stress conditions occur when the antioxidant defense in the body is unable to counteract the production and aggregation of reactive oxygen species (ROS). The previous literature has shown that phytochemicals and other natural products not only scavenge oxygen free radicals but also improve the expressions of cellular antioxidant enzymes and molecules. Consequently, these products protect against ROS-mediated cellular injury. This review aims to give an overview of the most relevant data reported in the literature on polyphenolic compounds, namely, gallic acid, resveratrol, quercetin, kaempferol, mangiferin, epigallocatechin, and pinocembrin, in terms of their antioxidant effects and potential protective activity against ischemic stroke.

## 1. Introduction

**Figure 1:**
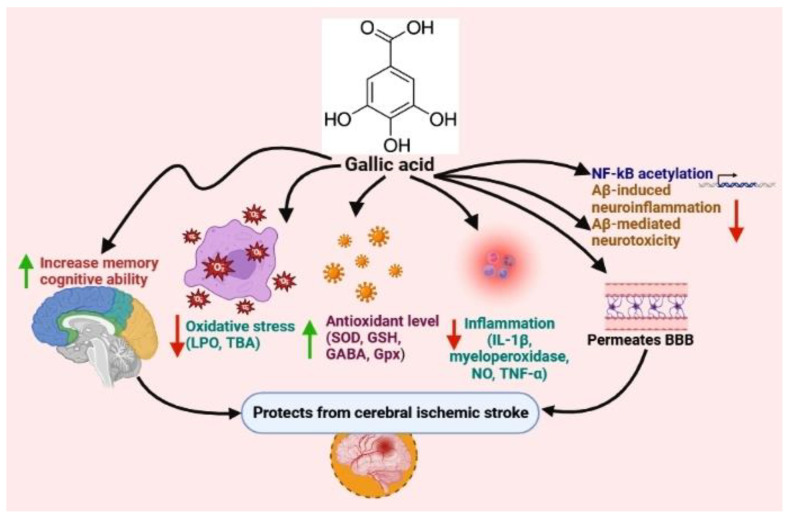
Effects of gallic acid on ischemic stroke (image created using biorender.com (accessed on 21 October 2022)).

Stroke is a cerebrovascular disease, mainly caused by atherosclerosis. It interrupts the supply of blood, which, in turn, causes an oxygen deficit [1]. The acute symptoms of stroke include dizziness, weakness, nausea, aphasia, hemiplegia, a loss of coordination, and a loss of vision. Stroke is the second leading cause of death in the world and one of the major causes of infirmity in adults, particularly in developing (12.8%) and developed countries (8.7%) [2,3]. Studies have also revealed an association between oxidative stress and various risk factors of ischemia stroke (IS), including cigarette smoking, in addition to hypertension, diabetes mellitus, hyperlipidemia, and obesity. Consequently, there is a huge number of relevant studies that have assessed the disease ontology and explored effective diagnostic measures and therapies. Some researchers have reported the positive effects of nutrient antioxidants on ischemic stroke [4,5,6]. Polyphenols are antioxidants that fight cancer. In pathological conditions, such as cancer, polyphenols are powerful antioxidants that mitigate oxidative stress. Free radicals can be scavenged by polyphenols. Essentially, there are more conjugated systems, aromatic rings, and hydroxyl groups in a different part of the molecules [7]. Humans consume these compounds in foods, such as fruits, cereals, and vegetables. Polyphenols can also prevent degenerative diseases. Research on polyphenols has been delayed due to their complex structure. Polyphenols are the most common antioxidants in our diet [8,9,10]. The action of these molecules is to inhibit oxidative change in low-density lipoprotein, which is the basic mechanism of endothelial lesions in atherosclerosis. Researchers have discovered that polyphenols are good for cardiovascular diseases, osteoporosis, neurodegenerative diseases, cancer, and diabetes [11]. In the current review, we aim to discuss the recent advancements in neuroprotection against IS using a wide range of polyphenols and to draw out their potential mechanism of action. We discuss, in particular, the polyphenols (gallic acid, resveratrol, quercetin, kaempferol, mangiferin, epigallocatechin, and pinocembrin) used in protecting the heart against IS and the related signaling pathways and mechanisms of recovery, highlighting the role of polyphenols. Figure 1 shows the basic structures of some common neuroprotective polyphenols.

## 2. Methodology

A literature search was carried out using a variety of databases, such as Scopus, Web of Science, PubMed, and Google Scholar, and the keywords utilized for this literature search were polyphenolic compounds, namely, gallic acid, resveratrol, quercetin, kaempferol, mangiferin, epigallocatechin, and pinocembrin, with oxidative stress associated with ischemic stroke. We excluded a few mechanisms that are not associated with oxidative stress associated with ischemic stroke.

## 3. Decreased Blood Flow Contributes to the Pathogenesis of Ischemic Stroke

Evidence from Mayhan et al., 2022, suggests that dysfunctional potassium channels may play a role in the etiology of vascular abnormalities and behavioral/cognitive disorders in the brain. Changes in the responsiveness of cerebral arterioles in response to the activation of essential vasodilator mechanisms may underlie the observed anomalies in brain function and, hence, impact the control of cerebral blood flow in response to metabolic demand variations (neurovascular coupling) [12]. Basal tone and variations in the diameter of cerebral arteries/arterioles and, hence, cerebral blood flow have been demonstrated to be regulated in response to several stimuli through ATP-sensitive potassium channels (KATP) and calcium-activated potassium channels (BK) [13]. The smooth muscles of blood vessels, including the arteries and arterioles of the brain, have been found to contain KATP channels [14]. After cerebral ischemia/reperfusion injury and in a number of other disease conditions, investigations have demonstrated that the dilation of cerebral arterioles in response to the activation of KATP channels changes. Basal tone in the cerebral arteries and arterioles can be modulated by BK channels. Dilating cerebral blood vessels in response to various agonists/physiological stimuli is mostly dependent on the activation of BK channels. The regulation of cerebral blood flow may be severely compromised if cerebral arteries are unable to respond normally to KATP and BK channel activation, especially under conditions of elevated metabolic demand. Impaired responses of cerebral arterioles to eNOS- and nNOS-dependent agonists could be attributed to an increase in oxidative stress. Large cerebral and coronary arteries are particularly susceptible to the negative effects of oxidative stress on K^+^ channel activity [15]. Superoxide is a critical modulator of diet-induced hyperhomocysteinemia-related cerebral vascular dysfunction and vascular hypertrophy. These results suggest that mild hyperhomocysteinemia may be an independent risk factor for cerebrovascular disease and ischemic stroke, and they may help provide a molecular basis for these observations in clinical practice. Future efforts to prevent the cerebral vascular consequences of hyperhomocysteinemia may target superoxide-dependent pathways, together with homocysteine-lowering medications, such as folic acid supplements [16]. Increased superoxide production from NAD(P)H oxidase activation is one mechanism by which aging reduces the eNOS-dependent responsiveness of cerebral arterioles and reduces oxidative stress [17]. Ischemic stroke results from a combination of factors, including oxidative stress, blood flow in the cerebral area, and dilated cerebral blood vessels [15].

Brain neutrophil infiltration and ischemia damage are both reduced in animals lacking ICAM-1 or treated with techniques that block ICAM-1 [18]. Additionally, blocking E-selectin is linked to better neurological outcomes [19]. When the brain suffers from ischemia, neutrophils are one of the earliest types of leukocytes to arrive on the scene. In addition to generating cytotoxic chemicals, neutrophils can clog blood vessels, reducing blood flow to the brain during reperfusion and, thus, worsening brain I/R injury. There have been a number of studies showing that preventing neutrophil infiltration into the brain reduces I/R harm [20]. The brain’s resident immune cells, called microglia, play a crucial role in regulating homeostasis and the immune response. There is evidence to suggest that there are two ways in which phagocytosis, the production of neuroinflammatory mediators that are harmful to cells, and activated microglia contribute to brain I/R injury. Damaged neurons, infiltrating leukocytes, activated astrocytes, microglia, and endothelial cells all contribute to the production of cytokines/chemokines in the aftermath of brief focal cerebral ischemia [21]. By activating microglia, upregulating the production of adhesion molecules, and driving pro-apoptotic signaling, pro-inflammatory cytokines, such as IL-1, TNF-, and IL-6, all contribute to brain I/R injury [22]. By contrast, anti-inflammatory cytokines, such as IL-1ra, IL-4, and IL-10, reduce pro-inflammatory cytokines and their receptor expression and downstream signaling to dampen inflammation after an ischemia event. There is an uptick in pro-inflammatory cytokines and chemokines, pointing to a potential for vascular inflammation [23]. This reduction of blood flow increases vascular inflammation and leads to ischemic stroke.

## 4. Oxidative Stress and Stroke

The central nervous system (CNS), microglia, and astrocytes are key sources contributing to the generation of reactive nitrogen species (RNS) and ROS, which regulate synaptic and nonsynaptic transmission between neurons and glia [24,25]. ROS and RNS stimulate the long-term potentiation of synaptic transmission, essential for memory. Moreover, studies have indicated age-related changes in superoxide in regulating synaptic ductility, learning, and forming memories. The brain is known to be at high risk following an increase in RNS and ROS caused by decreased neuron antioxidant enzymatic activity and (1) increased peroxidizable lipid concentration, (2) O_2_ consumption, and (3) iron levels, which act as pro-oxidants, inducing oxidative stress under pathological conditions [26,27]. To that end, it has been reported that the production of ROS has a significant impact on the brain on exposure to ischemic attack and reperfusion. 

However, the three major routes of physiological ROS production, in general, remain significant during a stroke (Figure 1). The glycolytic pathway and Krebs cycle are responsible for the generation of these reduced coenzymes, which undergo oxidative phosphorylation to generate ATP molecules [28]. However, with a decreased oxygen supply, the metabolism slows down the electron transport chain (ETC) while enhancing the formation of superoxide ions from complexes I and III [29]. Additionally, mitochondria absorb the Ca^2+^ ions entering the neurons, causing the depolarization of the membrane and the impairment of ETC, which leads to a higher production of free radicals and the production of ATP. The combination of Ca^2+^ and ROS assists in the opening of the mitochondrial permeability transition pore (MPTP), and the resulting membrane leakage causes the energy deprivation and complete depolarization of mitochondria. MPTP may further disrupt the mitochondria, resulting in the production of ROS, cytochromes, and Ca^2+^ in the cytosol, which causes cell damage and apoptosis [30,31,32]. During neuronal ischemia, it is challenging for mitochondria to maintain a sufficient level of ATP, as the cell membrane ion pumps use an extensive amount of ATP to counteract the influx of Ca^2+^ and Na^+^ mediated by the *N*-methyl-D-aspartate (NMDA) receptor [33,34,35]. ROS oxidizes the thiol groups present in the adenine nucleotide transporter aggravated by the ROS-mediated consumption of GSH, and this impairs the movement of ATP from the mitochondria into the cytosol. Consequently, a vicious cycle is formed between the increased ATP demand and the reduced capacity for production and delivery, resulting in a decrease in energy, membrane ion flux, and cell death.

Free radicals have a high reactivity and a short half-life, making it difficult to measure them directly. There is an indirect way to demonstrate the production of free radicals, which is by measuring the products of a reaction between free radicals and other molecules, including DNA, lipids, proteins, and antioxidant levels [36]. Rodents, such as rats and mice, are commonly used laboratory species for research on brain ischemia. Their cranial circulatory anatomy is similar to that of humans, their physiological factors are easy to control, and histopathology enables analyses of ischemic pathogenesis and tissue infarction. Numerous experimental and clinical observations in various animal studies have indicated a higher free-radical production during all forms of ischemic injury (Table 1). However, there are limited data for such a correlation in humans due to methodical issues in measuring free radicals.

## 5. Effects of Polyphenols on Stroke

The dietary consumption of polyphenols from different sources of plants provides protection against the morbidity and mortality caused by cardiovascular diseases. Polyphenols from different plant sources provides protection against stroke in humans, animals, and in vitro studies [89]. Polyphenols have different pharmacological and biochemical effects. Some polyphenols have anti-inflammatory, antioxidant, and anti-proliferative effects. Oxidative stress plays an important role in cerebral ischemia. Polyphenols provide protection against neurodegenerative diseases with cerebral ischemia by reducing ROS and apoptosis, thereby acting as therapeutic agents against stroke [90]. Polyphenols are found in plant products, and they help in the defensive response against various kinds of stresses, including physical damage and ultraviolet radiation. It has also been observed that phenolic antioxidants inhibit the oxidation of lipids and other molecules, which helps to provide protection against free radicals [91,92,93]. Moreover, the type of conjugate and the polyphenol structure can determine the antioxidant capability. This might be the reason behind the better performance of particular polyphenols in scavenging superoxides, while others can scavenge highly reactive radicals, such as peroxynitrite, derived from oxygen. Certain polyphenols can chelate iron and possibly prevent the free-radical formation caused by iron. In the last decade, researchers have taken a keen interest in the potential neuroprotective effects of polyphenols, such as grape and wine polyphenols, against cerebral ischemia [94,95]. Polyphenols play an important role in providing protection against ischemic stroke, as they protect neurons by decreasing oxidative stress through the inhibition of LPO and NO and by decreasing inflammation [96]. Polyphenols reduce vascular risk factors, such as atrial fibrillation, during a stroke. Moreover, they protect the brain by augmenting different mechanistic pathways; for example, honokiol has been found to have anti-thrombotic effects [97]. Polyphenols decrease the production of ROS by inhibiting oxidase, reducing superoxide production, inhibiting the formation of OxLDL, proliferating VSMC, inhibiting migration, reducing platelet aggregation, and providing protection against mitochondrial oxidative stress. Therefore, polyphenols provide protection against ischemic heart disease and stroke [98].

In preclinical models, when polyphenols are administered after the induction of stroke, they exert neuroprotective actions, delaying the progress of brain damage, as well as the recovery of stroke [99,100]. Polyphenols exhibit their neuroprotective effects at the mechanistic level by acting on various targets at the same time. These compounds are strong antioxidants, with hydroxyl groups and neutrophilic centers functioning as ROS scavengers and metal chelators. Certain polyphenols can also initiate transcription factors associated with antioxidant-responsive element pathways, including erythroid 2-related factor 2 (Nrf2) [101,102,103]. Therefore, they promote the activity of antioxidant enzymes, such as superoxide dismutase (SOD), heme oxygenase-1 (HO-1), catalase, glutathione reductase, and glutathione-S-transferase. Various polyphenols can interact with pro-apoptotic (Bax and Bad) and anti-apoptotic (Bcl-2 and Bcl-XL) members of the Bcl-2 family, p53, mitogen-activated protein kinases (MAPKs), and protein kinase B (AKT) [104,105,106,107]. The previous literature has shown that polyphenols can modulate the nuclear factor kappa-light-chain-enhancer of activated B cells (NF-kB), the Toll-like receptor (TLR), and arachidonic acid pathways. This decreases the formation of tumor necrosis factor α (TNF-α), interleukin (IL)-1β, IL-6, IL-1, and IL-8, along with cyclooxygenase-2 (COX-2), inducible nitric oxide synthase (iNOS), and nitric oxide (NO) [101,108,109]. 

### 5.1. Gallic Acid

Gallic acid (GA) is largely available in the free form or bound form as a derivative in various foods, including nuts, grapes, tea, honey, berries, fruits, and vegetables. It has been used in a wide range of applications in healthcare and exerts beneficial effects on the inhibition of stroke, cardiovascular disorders, Alzheimer’s disease, and Parkinson’s disease [54,90,110]. GA also has markedly increased activity to scavenge reactive oxygen species. GA decreases oxidative stress with increased antioxidant levels and attenuates the markers involved in inflammation [111]. This is largely due to the hydroxyl groups on the phenolic ring and intramolecular hydrogen bonds, causing a decline in the bond dissociation of the hydroxyl group in phenolic compounds [112,113]. 

The neuroprotective effects of GA are based on their ability to permeate the blood–brain barrier, directly scavenge the pathological concentration of ROS and RNS, and chelate transition metal ions. Due to this scavenging activity and the activation of key antioxidant enzymes in the brain, GA has been shown to break the vicious cycle of oxidative stress and tissue damage. Researchers are showing a huge interest in the potential effects of GA on stroke, as well as on improving memory, learning, and general cognitive ability. The latest evidence indicates that the compound may exhibit strong actions on mammalian cognition while reversing age-related degradation [110,114,115]. GA attenuates neurobehavioral activities by decreasing inflammation via decreases in interleukin-1β, myeloperoxidase activity, nitric oxide, and tumor necrosis factor-α; increases in antioxidant activities and glutathione levels with reduced oxidative stress; and decreases in apoptotic levels by attenuating caspase 3 levels [116]. Gallates can easily permeate the BBB, scavenge free radicals, and chelate metal ions, thereby exerting their antioxidant activities. The supplementation of gallic acid further improves the neuro-motor functions that degenerate during psychosis. At the mechanistic level, the compound lowers lipid peroxidation, the levels of dopamine, and inflammatory signals with enhanced GABA and GSH synthesis [115]. 

GA may have significant positive effects on reducing the level of thiobarbituric-acid-reactive substances, retrieving SOD, GSH, and CAT activity levels, and it has been found to protect the brain of rats under oxidative stress promoted by sodium fluoride [117]. Mansouri et al. showed that the oral administration of GA could substantially improve passive memory and thiol and GPx contents while reducing the concentration of MDA in the hippocampus and the striatum of Wistar rats treated with 6-hydroxydopamine [118]. Their study’s findings indicated that the stimulation of GA could enhance cerebral antioxidant defense, which is an effective approach to treating patients suffering from a stroke. The compound has also been reported to show significant effects in inhibiting NF-κB acetylation and in suppressing Aβ-induced neuroinflammation and Aβ-mediated neurotoxicity on BV-2 and Neuro-2A cells in vitro [119]. Overall, GA shows neuroprotective effects on different in vitro cell lines or in vivo animal models (Figure 1).

### 5.2. Resveratrol

Resveratrol (RS) (3,4′,5-trihydroxystilbene; C_14_H_12_O_3_) is a polyphenolic phytoalexin present in grapes, peanuts, berries, and wines. In addition, its antioxidant properties show anti-inflammatory, anti-apoptotic, and anticancer capacities [120,121,122]. It can also ameliorate kainate-induced excitotoxicity, thereby proving its neuroprotective benefits. Furthermore, the compound may enhance histological and behavioral parameters following acute CNS injuries, such as stroke [123,124,125].

Studies have shown an association between RS-mediated decreases in the levels of neuronal MDA and high levels of antioxidant enzymes and compounds, including SOD and glutathione (GSH), respectively [126,127,128]. HO-1, an antioxidant enzyme, particularly acts as a key factor in RS-mediated neuronal protection following reperfusion after an ischemic attack [129]. Moreover, a previous study found that, in the cortical neurons of a cultured mouse, treatment with RS induced HO-1 expression [130]. A study on the cortical neurons of a cultured mouse also showed that RS treatment protects against exposure to glutamate, while an HO-1 inhibitor abolished this protection [131]. Additionally, it demonstrated that mice knocked out by HO-1 were unable to exert the neuronal protection provided by the RS pretreatment after transient focal ischemia. Furthermore, RS treatment led to an increase in antioxidant transcription factor Nrf2 in the nucleus, resulting in reduced oxidative stress and the oxidation of mitochondrial proteins following spinal cord ischemia (SCI) in rodents [132].

**Figure 2:**
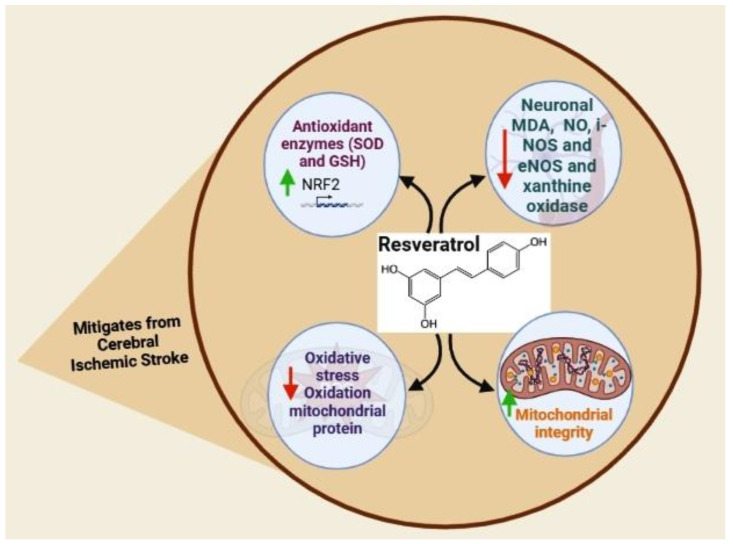
Effects of resveratrol on ischemic stroke (image created using biorender.com (accessed on 21 October 2022)).

A subsequent study supported these results, showing that RS treatment leads to a decrease in the levels of MDA while preserving mitochondrial integrity following SCI in rabbits [133]. Another study of RS treatment of SCI in rats showed increased levels of GSH along with decreased levels of NO and MDA and the activity of xanthine oxidase [134]. This study also indicated that RS treatment improves the basilar arterial lumen diameter and endothelin-1 activity [134]. In another study, resveratrol protected brain tissue against an increase in superoxide anion and against decreased vascular function dependent on endothelial nitric oxide synthase and neuronal nitric oxide synthase. Resveratrol’s antioxidant effects suggest that it could be used as a treatment for preventing neurocognitive decline and cardiovascular complications associated with type 1 diabetes [135]. Resveratrol therapy in diabetic rats improved eNOS- and nNOS-dependent cerebral artery reactivity, reversing the effects of type 1 diabetes. Further, resveratrol has been shown to counteract the rise in superoxide anion baseline levels associated with type 1 diabetes. These results suggest that resveratrol may have significant therapeutic promise for managing cerebrovascular dysfunction associated with type 1 diabetes [136]. Several key pathways controlling inflammation, mitochondrial function, oxidative stress, and cell death synergistically mediate the beneficial effects of RS (Figure 2).

### 5.3. Quercetin

Quercetin (3,3′,4′,5,7-pentahydroxyflavone) is a naturally found dietary flavonol belonging to a broad group of polyphenolic flavonoid substances. Quercetin has numerous beneficial properties, such as anticancer properties and the modulation of resistance to multiple cancer-related drugs, along with anti-inflammatory, anti-atherosclerotic, anti-thrombotic, and antihypertensive effects and the capacity for human endurance exercise [137,138,139,140,141,142]. An effective concentration of quercetin depends on permeability through the BBB and bioavailability to reach the neurons and exhibit protective effects. It has also been observed that liposomes carry quercetin to facilitate the activity of antioxidant enzymes and edema inhibition [143]. Schultke et al., in their study, showed a significant improvement in motor function in a rat model following acute traumatic spinal cord injury supported by a daily administration of quercetin (25 μmol/kg) [144]. This administration also resulted in a significant recovery from pathological conditions linked to brain trauma after the inhibition of myeloperoxidase activity in a rat model. Quercetin dihydrate has also been shown to protect transient middle cerebral artery occlusion (MCAO) rats against cerebral ischemic neuronal damage and upregulate antioxidant status [145]. 

Sirving et al. examined quercetin, and the result demonstrated the inhibition of the oxidative modification of LDL and a reduction in the cytotoxicity of LDL on the vessel wall [146]. Furthermore, in in vitro studies, flavonoids have been shown to reduce the aggregation of platelets, possibly through the inhibition of lipoxygenase and cyclooxygenase activity. They concluded that the long-term intake of quercetin may protect against stroke [147]. Lin et al.’s study suggested a prolonged survival effect of quercetin on heatstroke in rats by alleviating excessive hyperthermia and myocardial injury [58]. The anti-lipid peroxidative, antioxidant, and anti-inflammatory properties of quercetin contribute to its protective effects. Gidday et al. showed that, with the inhibitory effects of MMP-9 activity, quercetin reduced the disruption of the BBB during focal ischemia [148]. These results suggest the potential role of quercetin in protecting patients with focal ischemic stroke against neuronal injury.

**Figure 3:**
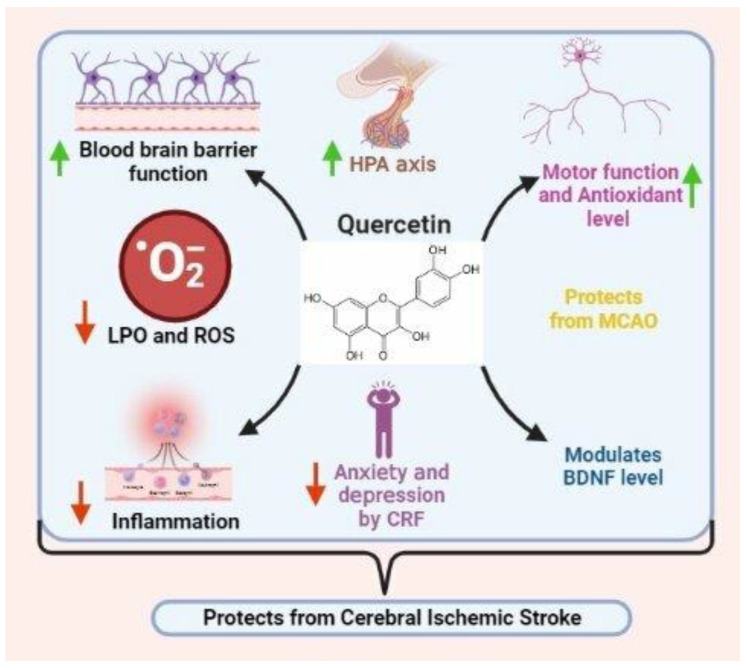
Effects of quercetin on ischemic stroke (image created using biorender.com (accessed on 21 October 2022)).

In a study with animals subjected to water immersion restraint stress, it was shown that quercetin treatment normalizes an elevated hypothalamic–pituitary–adrenal (HPA) axis. It also reverses the effects of anxiety and depression induced by a corticotrophin-releasing factor (CRF). The ability to modulate the level of BDNF further contributes to quercetin’s antidepressant-like activity, thereby making it a potential candidate for antidepressant drug therapy [149] (Figure 3).

Overall, studies have reported the cytotoxicity of quercetin based on its formation of toxic oxidation products, particularly orthoquinone (quercetin–quinone, QQ), during antioxidant activities. However, further research remains necessary to confirm the potential role of quercetin as a therapeutic agent in acute conditions, including advanced chronic neurodegenerative diseases and stroke.

### 5.4. Kaempferol

Kaempferol (3,5,7-trihydroxy-2-(4-hydroxyphenyl)-4H-1-benzopyran-4-one) is one of the most widely adopted aglycone flavonoids. The antioxidant and anti-inflammatory activities of this compound have been observed in various diseases, including diabetes, carcinogenesis, asthma, and encephalomyelitis [150,151,152,153,154,155,156]. Furthermore, KFL is effective in scavenging free radicals and preserving antioxidant functions, which delays the onset and progression of neurodegenerative disorders [157]. With an ability to cross the BBB, KFL exhibits a potential protective effect. It also acts as a potential dietary supplement, which is attributable to its multi-target property that prevents and treats neurodegenerative diseases [157]. 

An evaluation of kaempferide-7-O-(4″-O-acetylrhamnosyl)-3-O-rutinoside (KPF-7-O-rutinoside) was carried out in an in vivo MCAO ischemic model. The results illustrated the capability of the compound to reduce the neurological deficit through the inhibition of oxidative stress, the reduction of MDA levels, and the elevation of GSH-PX and SOD activities [158]. Additionally, indicating its neuroprotective, anti-inflammatory, and antiapoptotic effects, KPF-7-O-rutinoside inhibits NF-kB p65 phosphorylation; the levels of ICAM-1, COX-2, iNOS, and BAX2; and caspase-3 and -9 cleavage. Carmen López-Sánchez et al., in their study, showed that KFL exerts protective effects against nitrosative/oxidative-stress-induced ischemia/reperfusion brain injury following transient focal or global cerebral ischemia in experimental model animals [61].

**Figure 4:**
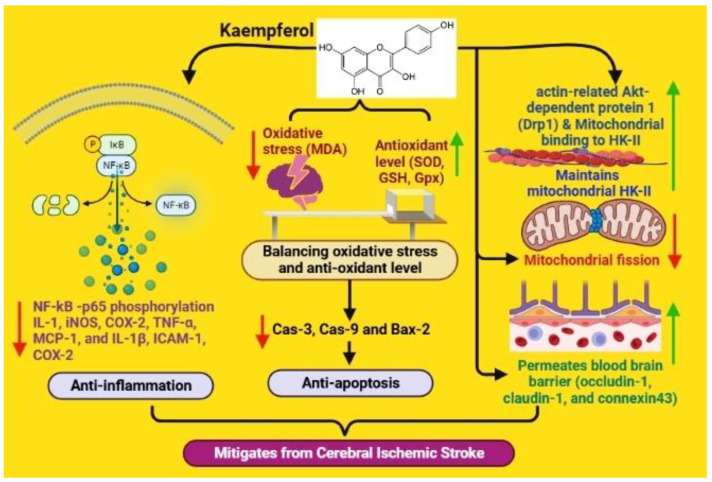
Effects of kaempferol on ischemic stroke (image created using biorender.com (accessed on 21 October 2022)).

According to Cheng et al., KF reduces the production of several pro-inflammatory and inflammatory proteins in brain tissue, such as IL-1, iNOS, COX-2, TNF-α, MCP-1, and IL-1β. In the mouse brain, it protects BBB integrity and increases BBB-associated proteins (occludin-1, claudin-1, and connexin43) [159]. Ischemic stroke is often associated with mitochondrial dysfunction. Wu et al. showed that KF inhibits mitochondrial fission and maintains mitochondrial hexokinase II (HK-II) to protect the neurons from ischemic injury. It also inhibits the activation of actin-related Akt-dependent protein 1 (Drp1) to promote the mitochondrial binding of HK-II [62]. This further suggests the modulation of Drp1 phosphorylation as a potential strategy for neuronal mitochondrial integrity protection and ischemic stroke treatment (Figure 4). 

### 5.5. Mangiferin

**Figure 5:**
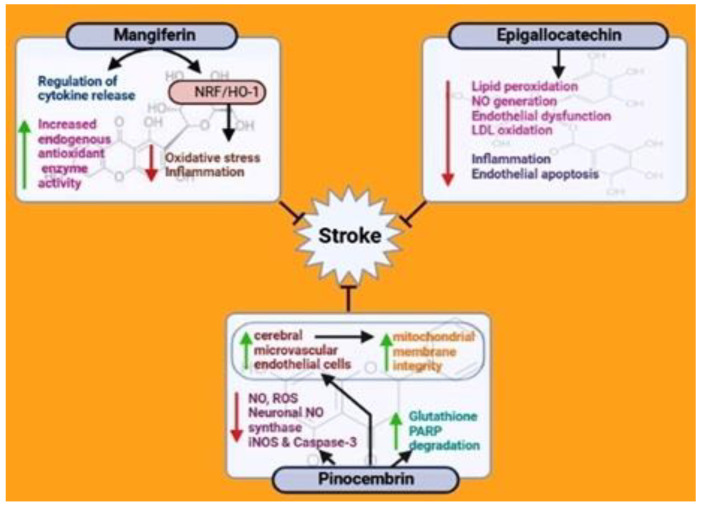
Effects of mangiferin, epigallocatechin, and pinocembrin on stroke.

Mangiferin (MFN) is extracted from mango, Iris unguicularis, and it belongs to the family Gentianaceae. It was first identified as a type of xanthone derivative and C-glucosylxanthone (2C-β-D-glucopyranosyl-1,3,6,7-tetrahydroxyxanthone). Attributed to its unique structure with glycosidic linkages, MFN exerts potential antioxidant properties with an ability to scavenge free radicals. The compound also possesses anti-inflammatory and anti-apoptotic activities, which protect against several organ injuries caused by different factors. The anti-diabetic and anticancer properties of MFN contribute to the regulation of inflammation, apoptosis, and cell proliferation, along with the mediation of immunomodulation [91,160,161]. Additionally, studies have reported the effects of MFN on the activation or repression of various signaling pathways, including the NF-κB, Nrf2/HO-1, and mitochondria-dependent pathways, and a component of the innate immune system, the NLRP3 inflammasome [162]. In terms of the CNS, previous studies have shown the positive effects of MFN in attenuating ischemic brain injury through the regulation of inflammatory cytokine release and the upregulation of endogenous antioxidant enzyme activity and Nrf2/HO-1 signaling cascade expression [71] (Figure 5). According to studies by Marquez et al., the compound minimizes inflammation and oxidative damage in rats using a stress model. They also reported the targets of MFN, such as catalase activity, lipid peroxidation, increased pro-inflammatory mediator levels, and a higher activation of NF-κB [68]. Moreover, the anti-inflammatory functions of MFN are similar to those of glucocorticoid, reducing capillary permeability and showing a protective effect against myocardial ischemia/reperfusion injury in rats. These studies suggest the role of mangiferin in protecting against cerebral I/R injury.

### 5.6. Epigallocatechin

The oral administration of EGCG in vivo has been reported to decrease lipid peroxidation product levels while increasing enzymatic and non-enzymatic antioxidant levels [163]. A previous study also observed a complete modification of the damaging action of AlCl3 on the activity of SOD and significant enhancements in the activities of glutathione peroxidase, cytochrome C oxidase, and acetylcholinesterase [164]. 

Lee et al. indicated that the free-radical scavenging potential and lipid peroxidation inhibitory activity of EGCG may reduce the occurrence of stroke, and they also found that the life span of stroke-prone hypertensive rats was prolonged [165]. In a study examining the protective action of green tea catechins, such as EGCG, on cerebral ischemic injury, Suzuki et al. (2004) suggested a potential action of the daily intake of EGCG in providing protection against cerebral ischemia damage [166]. Lim et al. (2010), in their study, used a rat model to evaluate the functional effects of EGCG on ischemic stroke. Their results showed the ability of EGCG to induce functional enhancements in the forelimbs in a rat model with ischemic stroke during the acute and subacute periods [75].

Various human, animal, and in vitro studies have also demonstrated that the intake of green tea can decrease the risk of stroke through potential biological mechanisms. It is important to note that the research community does not completely understand these mechanisms and that discrepancies exist [167]. Polyphenols can also modulate NO generation in the vascular endothelium and interfere with the mechanisms causing inflammation and endothelial apoptosis. Consequently, they prevent endothelial dysfunction, which plays a key role in the pathogenesis of cardiovascular disorders, such as stroke [168]. Studies have reported the ability of flavonoids to reduce LDL oxidation (Figure 5). Theanine, a tea extract, has also been observed to have a similar inhibition effect. Theanine and other tea compounds take up the active oxygen species that cause significant cell damage [169]. Moreover, tea consumption should be encouraged, as it can potentially contribute to stroke prevention.

### 5.7. Pinocembrin

Stroke survivors are often left with long-term, neurologic defects. Over the last decade, numerous research findings have shown the protective effects of pinocembrin on cerebral ischemic injury, with a wide therapeutic potential [170,171]. In both in vitro and in vivo studies, pinocembrin exhibited neuroprotective, anti-oxidative, and anti-inflammatory properties [83]. It also exhibits protective effects in rats with ischemic stroke. Such study results suggest the promising use of pinocembrin in novel, multiple-action drug therapy development. Shi et al. also reported an antioxidant effect of pinocembrin in 4-VO rats [84,172]. 

Pinocembrin is responsible for modulating excitatory and inhibitory amino acid concentrations and minimizing neuronal loss in rats following cerebral 4-VO ischemia in a dose-dependent manner (1, 5, 10 mg/kg). Regarding therapeutic potential, pinocembrin may still have a protective effect, even if the administration is set up to 6 h post-4-VO induction [172]. Consequently, it has a relatively wide application when compared to tissue plasminogen activator (tPA, 3 h), which is the drug used during ischemic stroke [173].

In vitro studies have shown the ability of pinocembrin to increase neuronal viability, reduce the release of lactate dehydrogenase, inhibit NO and ROS production, elevate the levels of glutathione, and downregulate neuronal NO synthase (nNOS) and iNOS expressions in primary cortical neurons exposed to oxygen–glucose deprivation/reoxygenation (OGD/R) [174]. Such findings demonstrate the neuroprotective effects of pinocembrin in vitro. This compound can also contribute to the regulation of mitochondrial function and apoptosis. In primary neurons subjected to OGD/R, pinocembrin reduced the expression of caspase-3 while increasing the degradation of PARP (Figure 5). The former has also been shown in tunicamycin-induced SHSy5y cells [175,176]. Pinocembrin-containing substances, such as Chinese propolis, have been shown to protect against ER-stress-induced neuronal toxicity [176]. A previous study demonstrated the ability of pinocembrin to protect the cerebral microvascular endothelial cells of cultured rats from OGD/R damage and to increase mitochondrial membrane integrity. These results suggest the neuroprotective and vascular protective effects of pinocembrin, supporting its therapeutic application in stroke [84].

## 6. Conclusions

Stroke is a devastating neurological disorder that accounts for a sizable portion of global mortality and disability. Stroke pathogenesis is intricately intertwined with other processes, such as excitotoxicity, inflammation, oxidative damage, ionic imbalances, apoptosis, angiogenesis, and neuroprotection [177]. Acute stroke triggers an ischemia cascade that ultimately results in the death of neurons and the permanent impairment of their functions. Ischemic stroke risk factors include atherosclerosis in the aortic arch. Atherosclerosis is a hardening of fatty deposits on the arterial inner wall (atheroma), which creates a rougher surface and increases the risk of clot formation and artery obstruction. Stroke can be caused by atherosclerotic alterations in the brain’s blood vessels. Atherosclerotic alterations in the carotid artery are indicative of a more widespread disease. Most ischemic strokes result from atherosclerotic plaque in the carotid bifurcation, and the severity of carotid stenosis is a good predictor of stroke incidence [178]. Pathophysiological processes, such as excitotoxicity, oxidative and nitrative stress, inflammation, and apoptosis, contribute to the complexity of brain injury after stroke [179]. Oxidative stress is one of the pathophysiologic states caused by stroke in the brain, leading to severe toxicity, inflammation, and apoptosis. Here, this review showcases the importance of phytochemicals and other natural products in scavenging free radicals to improve antioxidant enzymes and other key molecules that are required for normal cellular activities. The use of polyphenols can tremendously prevent cell damage and edema during cerebral ischemic injury. While there is no argument about the deleterious effects and detrimental contribution of free radicals to lesion progression after ischemic stroke, the clinical efficacy of antioxidants in this setting remains unclear, indicating the importance of further investigations of the cellular and molecular actions of polyphenols in neuroprotection. Here, we conclude that phytochemicals and other natural products, namely, gallic acid, resveratrol, quercetin, kaempferol, mangiferin, epigallocatechin, and pinocembrin, are involved in providing neuroprotection against IS via various cellular mechanisms, underscoring the need for further studies on these compounds to determine their healthcare benefits for humans.

## Figures and Tables

**Table 1:** Effects of polyphenols on stroke in animal studies.

**Name of the Polyphenol**	**Mode of Study**	**Major Findings**	**Refs.**
Resveratrol	Mouse and Rat	Oxidative stress inhibition, anti-inflammation, brain damage and apoptosis inhibition	[37,38,39,40,41,42,43,44,45,46,47,48,49,50,51]
Gallic acid	Mouse and Rat	Oxidative stress reduction, anti-inflammation	[52,53,54]
Quercetin	Mouse and Rat	Oxidative stress inhibition, MMP9 reduction	[55,56,57,58,59]
Kaempferol	Mouse and Rat	Oxidative stress inhibition, anti-inflammation, mitochondrial dysfunction suppression	[60,61,62,63,64]
Mangiferin	Mouse and Rat	Oxidative stress inhibition, anti-inflammation	[65,66,67,68,69,70,71]
Epigallocatechin	Mouse and Rat	Oxidative stress inhibition, anti-inflammation	[72,73,74,75,76,77,78,79,80]
Pinocembrin	Mouse and Rat	Oxidative stress inhibition, anti-inflammation	[81,82,83,84,85,86,87,88]

## Data Availability

Not applicable.
